# A systems thinking approach to understanding the challenges of achieving the circular economy

**DOI:** 10.1007/s11356-020-11725-9

**Published:** 2020-12-07

**Authors:** Eleni Iacovidou, John N. Hahladakis, Phil Purnell

**Affiliations:** 1 Division of Environmental Sciences, College of Health, Medicine and Life Sciences, Brunel University London, Kingston Ln, Uxbridge, London UB8 3PH UK; 2 College of Arts and Sciences, Center for Sustainable Development, Qatar University, P.O. Box: 2713, Doha, Qatar; 3 School of Civil Engineering, University of Leeds, Woodhouse Lane, Leeds, LS2 9JT UK

**Keywords:** Sustainable, Circular economy, Systems thinking, Value, Resource recovery, Challenges

## Abstract

Circular economy (CE) is extensively discussed around the globe. Presently, discussions are mostly concerned with the importance of achieving CE and the benefits associated therewith, with the various barriers surrounding its implementation being less debated. Understanding the context in which circularity can flourish is a prerequisite in building the capabilities to deal with the multi-faceted challenges that currently hamper progress in closing the material, component and product loops. In this study, we discuss the importance of systems thinking in understanding the way resource recovery systems operate, and in promoting deep transformational change. We suggest that transformational change needs to go beyond closing materials, components and products (MCPs) loops, and promote sustainability in the way resources are exploited, used and managed throughout the system. By adopting a system of systems approach, we postulate that there are five interconnected sub-systems that need to be considered for supporting transitions to CE, namely, resource flows and provisioning service; governance, regulatory framework and political landscape; business activities and the marker; infrastructure and innovation; and user practices. This holistic approach provides a useful means to cutting through systemic complexity, and focuses on the dynamics between processes, values and actors in the value chain, and their dependence on cultural, spatial and temporal characteristics. We conclude that a systems-based approach can build up the capabilities required to identify and understand persistent linear trends and, in turn, support forward-thinking and time investment in enabling sustainable transitions. This, in turn, can help to align priorities and transform our current practices, speeding up the process of closing the MCP loops in a sustainable manner.

**Figure:**
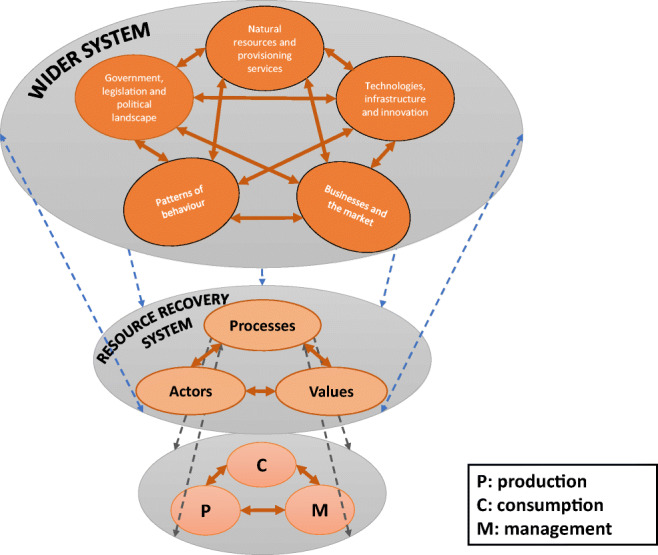
Graphical abstract

Graphical abstract

## Introduction

Circular economy (CE) is a concept that goes beyond waste reduction. It embraces the idea that materials, components and products (MCPs) should be designed and produced so that they can be restored, retained and re-distributed in the economy for as long as it is environmentally, technically, socially and economically feasible (Hahladakis and Iacovidou 2019). Repair, remanufacture, reuse and recycling processes can slow down or reduce additional negative value creation. They can minimise further degradation, or dissipation of MCPs environmental (e.g. water, energy), economic (e.g. costs of design, manufacture and distribution), social (e.g. labour intensity) and technical (e.g. properties, quality) values. Furthermore, retaining MCPs in the economy through such processes delays the point in time at which one, or all of these values, decay to the extent that they become ‘waste’ (Iacovidou et al. 2017b).

With too much focus placed on achieving CE, there is now a misconception that circularity is synonymous with sustainability. Whilst the concept of CE was conceived to promote resource efficiency and prevent waste, not all MCPs can promote sustainability by being forced into circularity. Given this large and growing problem, it is becoming gradually recognised that understanding the connections between production, consumption and management processes is urgently needed in assessing and monitoring progress towards sustainable transitions to CE. In response to this issue, a growing body of policy initiatives have emerged to promote sustainable consumption and production (Bengtsson et al. 2018; Koide and Akenji 2017; Vergragt et al. 2014). Notwithstanding the important contributions of these initiatives, not much progress has been achieved to date towards the integration of production-consumption-management processes. This is due to efforts being largely placed either at downstream ‘end-of-pipe’ processes of managing wastes or at upstream control of material throughput in the economy (Koide and Akenji 2017). This divergent focus emphasises the presence of several obstacles towards achieving CE that require further scrutiny.

A number of studies have attempted to analyse the scale and breadth of CE challenges. Some studies emphasised aspects in the environmental and economic domains of value (Ghisellini et al. 2016) placing little attention on interactions with the social and technical domains (Bengtsson et al. 2018; Korhonen et al. 2018a), whilst others have discussed CE implementation challenges from a supply chain management (Govindan and Hasanagic 2018), or an integrated materials perspective, underlining the disconnect between supply chain actors (Korhonen et al. 2018a; Velenturf et al. 2019). Even though, these approaches are beneficial in addressing some of the key issues around CE implementation, they are not cohesive in their approach to addressing the CE challenges. Several authors indicate that the lack of coherent approaches can be attributed to the lack of a broadly accepted definition of CE (Corona et al. 2019; Mayer et al. 2019). What’s more, Mayer et al. (2019) suggest that the CE concept is applied differently by the multiple stakeholders involved in the value chain, and is often contingent to their interests and values (Mayer et al. 2019). This lends itself to a plethora of reviews on CE and CE definitions that inconsistently take into account environmental, economic and social dimensions (Kirchherr et al. 2017). A cross-cutting and unifying approach is thus necessary to characterise the (un-)sustainable production, consumption and management processes.

Here, we explain that systemic thinking and practice at multiple levels—from personal to local, national and international—is key in identifying and overcoming barriers to sustainability in resource recovery systems, and elucidate how the lack of such an approach can create circularity mishaps. To postulate this idea, in the ‘Examples of CE ‘mishaps’ in resource recovery systems’ section, we present and discuss some well-established examples of resource circularity in disguise, highlighting their related counter effects. We then retrace the ontological and theoretical background of systems thinking approaches to explore the foundations and robustness of our approach and to bridge resources and waste management to engineering, policy science and transition management. Specifically, in the ‘A systems-based approach to realising transitions to CE” section, we (1) outline the features of different theories and ontologies on systems and sustainable development in a comparable fashion, in order to highlight their crossovers, and importance of their potential integration; and (2) develop an overarching systemic framework that can be used to conceptually assess the efficiency, barriers and drivers of existing and new ways of promoting resource recovery and circularity. We then use our framework in the ‘Using the ‘five levels of information’ to unpacking CE challenges’ section, to discuss some of the CE challenges that arise due to the existing conventional and persisting structures and processes, following an integrative analysis of our perspectives on the field (as evolved from our research) and assimilation of the global literature in our effort to validate our thinking. Finally, the study concludes with recommendations for further research in this area.

## Examples of CE ‘mishaps’ in resource recovery systems

Past efforts in recovering resources from waste have predominantly focused on energy recovery and recycling. Both energy recovery and recycling processes dominate public and private investment in waste infrastructure (Purnell 2019), as they are considered economically attractive waste management routes that reduce (but not eliminate) the immediate environmental and social costs of waste. Yet, neither of these processes promotes the retention of technical value of resources in the long-term.

For instance, energy recovery from waste prevents harmful materials from being disposed of to landfills and recovers some thermal value from waste that would otherwise be discarded. Nonetheless, it destroys perfectly functional MCPs, permanently removing their potential additional value from the economy (Bi et al. 2010; Huang et al. 2013; Peeters et al. 2014). Likewise, recycling is often assumed to result in net greenhouse gas (GHG) emissions savings due to its significant potential to reduce energy consumption compared to that used in the processing/ production of primary materials (Akenji et al. 2016). This is true for energy-intensive materials such as steel, aluminium or plastics, but the recycling of materials such as concrete, plasterboard, bricks and paint can often result in higher net GHG emissions compared to producing their primary counterparts (Cullen 2017; Fitzgerald et al. 2012; Lee et al. 2017; Turner et al. 2015; Turner et al. 2016). This highlights that the type and quality of materials recycled may considerably affect the net GHG savings of recycling, as does the sorting and reprocessing methods used, and the proximity/place of the recycling facilities (Bakker et al. 2014; Dahlbo et al. 2018; Elia et al. 2017; Hahladakis et al. 2018a; Huysman et al. 2017; Prendeville et al. 2014; Ramani et al. 2010; Reh 2013; Shahbazi et al. 2016; Winkler 2011). Moreover, almost all recycling methods require significant energy inputs. For example, recycling 1 t of aluminium requires 7.4 GJ (European Commission 2000); whilst this represents a > 90% saving over refining primary aluminium (Johnson 2015), it is still a very large amount of energy, on the same order as the annual electricity consumption of the average UK household. All of the above implies that energy recovery and recycling can in some cases be seen as linear options in disguise, maintaining practices that can be unsustainable in the long-term.

In a similar fashion, design and manufacturing practices can also impact on the fate of components and products introduced in the economy. Product designers often have no incentives to incorporate end-of-life (EoL) considerations into their products, but this is gradually changing. In actual fact, design for disassembly and recycling (i.e. avoiding mixed materials, composites, fixed joints that cannot be dismantled, modular design) has recently gained attention, particularly in the fields of plastic packaging (WRAP 2018) and construction (Hyams et al. 2018; Parliament UK 2018). Consumers and policy-makers are now questioning the amounts of packaging used and its potential for recycling (Iacovidou and Gerassimidou 2018), and even though plastic packaging cannot be presently entirely removed from the product chain due to its manifold functionality (e.g. lightweight, corrosion resistance, high thermal/electrical insulation and durability, marketability, traceability and communication) considerations for removing, or substituting some of the plastic packaging with other materials, have been undergoing. This has led to a growing demand for sustainable plastic packaging, which in turn has led to changes in the flexible (pouches) and multi-layer plastic packaging.

Polyethylene terephthalate-poylethylene (PET-PE) plastic pouches (non-recyclable) have increasingly been replaced with sustainable pouch alternatives made of 100% PE or polypropylene (PP) that are fully recyclable. Nonetheless, PE and PP cannot provide the sufficient oxygen barrier or the stiffness needed for products such as, milk powder, coffee, potato chips, fresh pasta and nuts (Mueller et al. 2012). As a result, introducing a layer of a barrier and stiffness enhancing material is needed to protect the contents of the polymer against UV radiation, oxygen and other gases. The most common barrier materials used are the ethylene vinyl alcohol (EVOH) and silicon oxide (SiOx) coatings. These materials are now commonly added to oriented polyethylene-light density polyethylene films (OPE-EVOH-LDPE) and baxially oriented polypropylene films and trays (BOPP-EVOH-BOPP and BOPP-SiO_x_-CPP) (Langowski 2008), leading to improvements in the barrier properties against oxygen or water vapour (Mueller et al. 2012). However, PP and PE plastic pouches and trays reinforced with EVOH and SiO_x_ coatings are shown to have a negative impact on the recycling process, due to the dispersion of these substances in the mix (Mepex Consult AS 2017). Although COTREP suggests that there is tolerance of > 5% EVOH in the PE and PP streams, EVOH and SiOx enhanced packaging that is increasingly introduced in the market could represent an important barrier to the recyclability potential of PE and PP recyclable materials (COTREP 2017; Mepex Consult AS 2017). This signifies that replacements in the plastic packaging components that promote the rationale of ‘design for sustainability’ are in fact limited by thresholds in their recyclability performance, which need to be better understood in order to avoid creating negative sustainability impacts. This is further supported by the increased focus on the introduction of bio-based plastic packaging.

A rising demand for bio-based plastics is suggested to lead to higher resource throughput as they require higher amounts of energy and water to produce than their conventional counterparts (Álvarez-Chávez et al. 2012). The lack of appropriate infrastructure for their management, and the public misconception that all bioplastics are biodegradable in the general environment (when it is in fact unknown how quickly they degrade (if at all) in the environment), may introduce contamination in the recyclable and organic waste streams and disintegrate efforts in avoiding littering at all costs. This highlights that the use of bio-based plastics can shift environmental burdens to other stages in the value chain rather than reduce their overall life cycle impacts (Hahladakis et al. 2018a; Iacovidou and Gerassimidou 2018). Better scrutiny over their imminent use is urgently needed, which emphasises that ‘design for recycling’ should not be achieved to the exclusion of ‘design for sustainability’. This is also true for components and products that are technically challenging to recycle, such as composite packaging or electronic components glued together. Products that contain different types of materials are difficult, or often impossible, to recycle. This is due to difficulties in separating the constituent materials from each other in order to recycle them (e.g. crisp bags).

‘Design for reuse’ has also been receiving increased attention. Extending the useful life of MCPs can bring about several environmental and socio-economic benefits. It can limit the amount of raw resources used, facilitate the provision of socio-technical benefits via job creation, and promote access to various MCPs by people with less resources (rreuse Unknown n.d.). But, even reuse has its trade-offs. For example, extending the product life of electric and electronic equipment (EEE) is effective in reducing materials and energy consumption upstream (at production stage), but it may contribute to more energy consumption downstream (e.g. at the use stage). Old products tend to consume more energy during their use, which may or may not offset the benefits of life extension (Truttmann and Rechberger 2006). Another example of component/product life extension is the use of plastic bottles filled with sand and food wrappers, as building units (e.g. walls, slabs, paver blocks) in developing contexts (Kumi-Larbi et al. 2018; Lenkiewicz and Webster 2017; Mansour and Ali 2015). Whilst this reuse model has significant environmental, economic and social benefits in communities that lack adequate waste management services (Cooper et al. 2011; Gorji et al. 2019), it can also present serious human health concerns due to additives behaviour (e.g. release to the surrounding environment and inhaled by the people) and mixture effects (Hahladakis et al. 2018b). This underlines that reuse must be considered on a resource-by-resource basis to ensure that intended multi-dimensional (i.e. environmental, economic, social and technical) benefits are realised, and overall life cycle impacts are reduced.

In the production-consumption systems, resource efficiency and dematerialisation had also affected resource throughput and management. For example, a reduction in the material and labour intensity of mobile phone making—a resource-efficient practice—has made phones affordable. Affordability has led to an increase in mobile phone demand and a consequential overall increase in the materials used for their production. The rapid development of the utility of mobile phones—from simple communication devices to multifunctional personal computers, cameras and social media terminals—has quickened their obsolescence, leading to shorter useful lives. The benefits of resource efficiency are now outweighed by the large amount of mobile phones produced, used and disposed unexploited worldwide. Similarly, the production of lightweight, fuel-efficient car by using aluminium reduces the ownership cost and leads to an increase in the travel rate (due to fuel-efficiency) and associated emissions (Abdoli et al. 2019), offsetting the expected benefits.

These are only but few examples that demonstrate the counterproductive effects occurring at the production, consumption and management levels and which may lead to unintended consequences, the so-called rebound effects (Abdoli et al. 2019). This is possibly because changes are either rushed into our system before they are well-understood, or occur in silos, leading to a rather fragmented pattern of solutions towards achieving the CE. These exemplify the need to view CE interventions that are conceived, developed and deployed for promoting the circularity of resources in a sustainable manner, as processes embedded in increasingly complex and interlinked systems.

## A systems-based approach to realising transitions to CE

### Systems thinking approaches and promotion of change

A number of theories, frameworks and strategies have been developed over the past decades to facilitate systemic thinking and modelling in an effort to promote sustainability. Some of the theoretical influences that are directly related to resource recovery systems include the cradle-to-cradle (McDonough and Braungart 2010), the performance economy (Stahel 2010), industrial ecology (Graedel 1994) and industrial symbiosis (Chertow 2000), a description of which can be found in our previous work (Iacovidou et al. 2017b). Whilst these approaches provide useful contexts and guidelines for improving resource efficiency and management at a sectorial and national economy level, they do not fulfil the need for systemic analyses that can provide a multi-dimensional perspective on the creation, destruction and dissipation of value in complex social, economic and political contexts. Their focus is on reducing material and energy throughput in the economy, overlooking the underlying social, political, economic and technical aspects.

Transition management addresses this gap, by implicitly acknowledging the role of society and technology in achieving sustainable change (Rotmans et al. 2000). Transitions management is a theoretical approach that stipulates that radical, socio-technical transitions are required for governments, individuals, communities and businesses, to aid the formulation of practicable strategies for their implementation and management (Rotmans et al. 2001). It goes beyond technological innovations to include institutional and sociocultural transformations in describing the challenges and dynamic nature of changing current paradigms, and to support strategic development(s) through a multi-level perspective (MLP) (Geels 2002). However, the implications of the proposed changes in the multiple value domains (i.e., environmental, economic, social, technical) are not implicit to the transitions management process. MLP views transitions as circular processes that result from the interplay of multiple dimensions operating at three levels (i.e. niches (radical innovations), socio-technical regimes and an exogenous socio-technical landscape) that interact with each other (Geels 2002; Geels 2011). It includes economics and science innovation as social processes shaped by institutions and structures. MLP can be a useful framework for understanding transitions, as it can highlight the dynamics and complexity of innovation. In other words, the use of the MLP can be particularly useful in understanding interventions and how interventions can be made, but is relatively weak in highlighting *where* interventions are needed in the wider resource recovery system.

Another framework that has emerged in the sustainability transitions arena is the Technological Innovation Systems (TIS). TIS focuses on understanding how specific technologies at the wider system can lead to socio-technical transitions, focusing on the identification of key processes, so-called functions, that need to be explored in relation to their drivers and failures (e.g. poorly working networks, institutional failures, infrastructure failures) (Bergek et al. 2008). According to Markard et al. (2012), this framework places attention to radical (and often more sustainable) innovations early during their development, which ensures their effective development and implementation (Markard et al. 2012). The TIS framework can be exceptionally useful in understanding the role of functions (i.e. processes involved in the system) in terms of their driving forces and blocking mechanisms in the wider systems, enabling as such the identification of key policy challenges (Bergek et al. 2008). At present, TIS has been used in analysing transitions to specific technologies, and its transposition to a resource recovery system perspective is yet to be grasped.

The framework for strategic sustainable development (FSSD), developed by Robert (2000), is another well-thought, structured method to cutting through systemic complexity and enabling transitions (Robèrt 2000). FSSD is comprised by a five-level model that includes the following steps: understanding the system; defining the visions via the use of a set of guiding sustainability principles to reduce vagueness and diffusion; strategizing the different ways of approaching the principle-defined vision; developing a strategic plan of concrete actions to achieve the desired vision; and using the necessary tools to monitor and support decision-making. In FSSD, stakeholders collaborate to develop a joint vision definition, which guides the co-creation process of possible strategic sustainable transitions (e.g. energy systems, transportation system). This process promotes social learning and co-creation that is guided and supported by backcasting (Broman and Robèrt 2017). Backcasting is a method where visions are defined and the actions required to achieve them are determined by working backwards (Quist and Vergragt 2006). In FSSD, backcasting can be used in combination with other tools to facilitate learning and analysis (e.g. modelling, simulation, life cycle assessment) (Broman and Robèrt 2017). Nonetheless, FSSD has presently been used in promoting the development of sustainable futures at corporate level; therefore, its operationalisation at a resource recovery system level remains to be explored (Broman and Robèrt 2017).

Backcasting in itself has also been used as a method of enabling systemic change. As backcasting is about desirable futures, it has been widely used in planning towards sustainability (Holmberg and Robert 2000), and for the development of sustainability pathways (Carlsson-Kanyama et al. 2008; Iacovidou and Wehrmeyer 2014; van de Kerkhof and Wieczorek 2005). Backcasting is useful in studying problems that are complex and involves persisting trends that contribute to the problems’ complexity, but supports decision-making in broader sustainability issues in the exclusion of the inherent complexity between different value dimensions.

Employing transition and backcasting frameworks is essential in identifying ways to achieve circularity and steering the course of action towards its realisation. Notwithstanding their importance in driving change, the development of a guiding method to understanding how resource recovery systems operate, according to contextual differences, is pivotal in orchestrating the entire process. To that end, transition management theory is acquaint with an important definition: that *transitions* are described as a set of *interconnected changes* that take place in different areas, such as technology, the economy, institutions, and behaviour and culture, ecology and belief systems (Rotmans et al., 2001). In this article, we use this notion of transitions to conceptualise resource recovery systems’ structure and develop an approach to streamline the process of gaining insights into their (resource recovery systems) functioning, painstakingly highlighting the main obstacles and challenges to achieving circularity.

### Developing a systems-based approach to understanding resource recovery systems’ complexity

In the study of Boardman and Sauser (2006), a system is defined as ‘a collection of entities and their interrelationships gathered together to form a whole greater than the sum of the parts’ (p.118) (Boardman and Sauser 2006). The Earth itself is such a system, which is subdivided into four main interconnected parts, known as sub-systems or spheres: the lithosphere, hydrosphere, atmosphere and biosphere. All activities, variability and change on the earth system depend on the interactions between its sub-systems that occur via the flows of materials and energy in complex biogeochemical cycles. In basic systems thinking terms, the sub-systems of a system exist and interact in a complex way within the system boundaries. System boundaries separate a system from its surroundings, much like the upper edge of the atmosphere that separates earth from its surrounding universe.

**Fig. 1:**
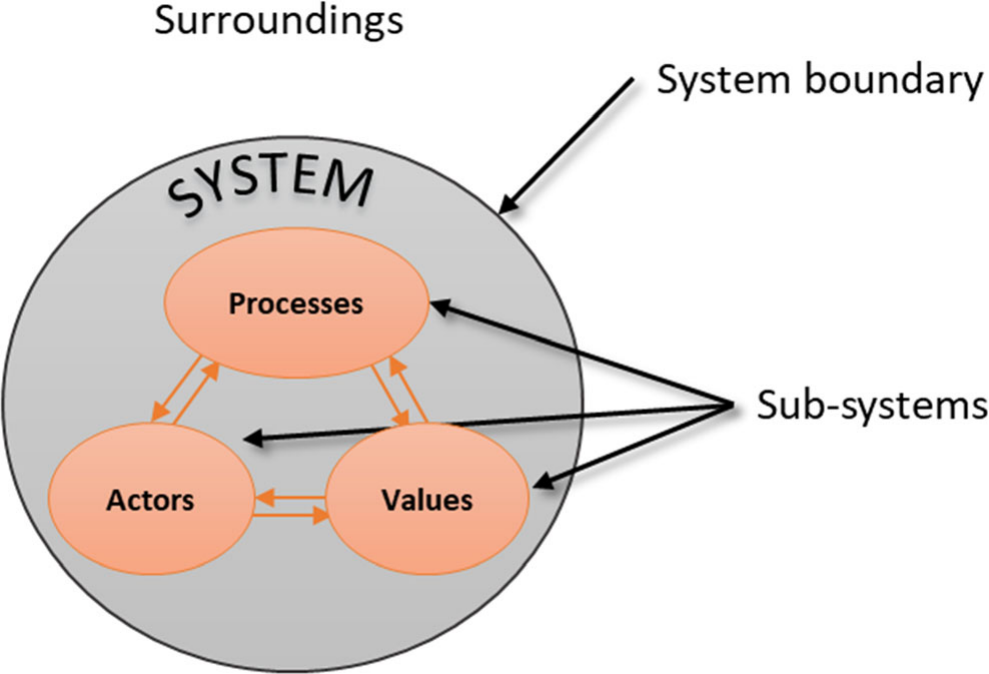
Representation of a resource recovery system. The system is separated from the surroundings via the system boundaries; within the system boundaries, there are three key ‘sub-systems’ that interact with one another

Here, we suggest that in resource recovery systems, the boundaries can be space-specific (e.g. city, country, ecosystem, organisation); resource-specific (e.g. material, component, product, energy, substance); process-specific (e.g. paper pulp manufacturing, plastic waste reprocessing); or, as is often the case, a combination of these. We also consider that any resource recovery system involves three core interconnected ‘sub-systems’ whose behaviour affects the entire resource recovery system, namely, the *processes*, *actors* and *values* that are functioning as a whole. These ‘sub-systems’, which are presented in Fig. 1, constitute the internal parts of a resource recovery system (i.e. are endogenous) and can affect the system’s properties and behaviour, but none of these (sub-systems) on its own has an independent effect on the resource recovery system. They are interconnected.

The ‘processes’ in the resource recovery system involve all stages of a resource’s flow in its various forms from production, to consumption and EoL management, which are by themselves separate sub-systems. In line with the law of conservation, matter cannot be created nor destroyed, but can be transformed from one form to another (e.g. crude oil to plastic, and plastic to energy and emissions). Therefore, to get an integrated view of the resource flows in a system, all inputs, outputs, stocks or sinks, leakages and hidden flows must be depicted by looking at the system as a whole, and never by looking at the production, consumption and management parts independently. This is because resource flows and transformations in a system occur as a result of the way the resource recovery system’s parts (i.e. production-consumption-management) interact. Flows are connected from one process to another, and the flow pattern is influenced by those who run the system, i.e. actors, and the pertaining factors that impact on value recovery, creation and dissipation.

The ‘actors’ are all the stakeholders involved in the resource recovery system, who are directly (e.g. manufacturers, retailers and waste management industry) and indirectly (e.g. government, NGOs) involved in the movement and processing of resource flows and the way the various processes are laid out and operated. They are in control of the ‘entry’ and ‘exit’ of resources (in same, or different forms) from the various ‘processes’, and are largely driven by their interests, as well as the socio-economic, political and technical processes that underlie resource flows. Understanding of the main socio-economic, political and technical processes that control resource exchanges (direct and indirect) and underlie the dynamics between stakeholders, can provide a sound grasp of their power relations established in the way production, consumption and management ‘processes’ interact (Iacovidou et al. 2020a). Power is intrinsic to human interaction, social organisation and the shaping of societal change, and therefore, understanding power dynamics is key to understanding what drives production and consumption patterns and creates barriers against attempts to make it sustainable (Fuchs et al. 2016). Insight into how actors interact and their power relations are key in identifying where potentially effective intervention points may exist in the resource recovery system.

The ‘values’ refer to the positive and negative impacts in the environmental, economic, social and technical domains as influenced by the respective processes, and the perceived needs, concerns and other considerations of stakeholders, including society—they represent the institutional settings. The selection of multi-dimensional values in a resource recovery from waste system is key in representing the different aspects of reality (from a multi-disciplinary, multi-sectorial perspective). These values are capable of providing critical insights to cause and effect relationships of resource recovery systems and reflect the potential of driving change. Values are represented by metrics which must be selected from all four domains of value (i.e. environmental, economic, social and technical). As a result, metrics aid the evaluation of resource recovery systems, which helps to identify the points where multi-dimensional value is captured, maintained, dissipated or could be created.

The processes, actors and values continuously interact and affect one another in numerous ways, and may vary depending on the spatial (i.e. cities, regions, countries) and temporal (i.e. present, future, different time zones) boundaries of the resource recovery system. In regard to the latter, a resource recovery system assessment can focus either on unravelling the persistence of critical obstacles to achieving sustainability in a specific point of time (static assessment), or on uncovering the emerging challenges and changes needed to enable transitions to sustainability over a specific period (dynamic assessment). In the surroundings of a resource recovery system, there are constantly intertwining ecological, economic, social, political and technological factors, i.e. the ‘drivers’ and ‘shapers’ of resource production, consumption and management. These exogenous factors arise from the interdependencies between the natural, social, economic and political systems, and can influence the multi-scale characteristics of the resource recovery system. In turn, this can induce several complexities in the synergies and trade-offs amongst its endogenous ‘sub-systems’ (i.e. processes, actors and values).

Following the transitions management theory, the synergistic relationship between the sub-systems (internal and external to the resource recovery system) may result in a set of interconnected changes that enable transitions to become realised. The notion of interconnected changes in systems theory can be associated with the concept of co-evolution. Co-evolution refers to the process where the interacting systems have a causal influence on each other’s evolution. Kallis and Norgaard suggest that many of the elements that matter in the social and natural worlds, such as resources, technologies, rules, beliefs, values and behaviours, are co-evolving, highlighting the interdependence between people and nature, which diffusedly affect the evolution of each other (Kallis and Norgaard 2010). This co-evolutionary process can be both mutually cooperative and competitive (Kallis and Norgaard 2010), which narrates the importance of synergism. Synergism can be defined as the combined interdependent effects produced by two or more sub-systems as a way to enhance the benefits of the outputs, a functional basis for the evolution of complex systems (Corning 1998). This synergistic effect relates not only to the functionality of systems (e.g. whether it is engineering, socio-technical or resource based) but also to processes (Loorbach 2010), stakeholder roles and involvement in maintaining and/or changing the system, and values (e.g. issues of concern, positive and negative environmental impacts, operational ability, performance qualities, stakeholders perceptions) (Geels 2004; Iacovidou et al. 2017a; Mitchell et al. 1995; Nielsen et al. 2015).

**Fig. 2:**
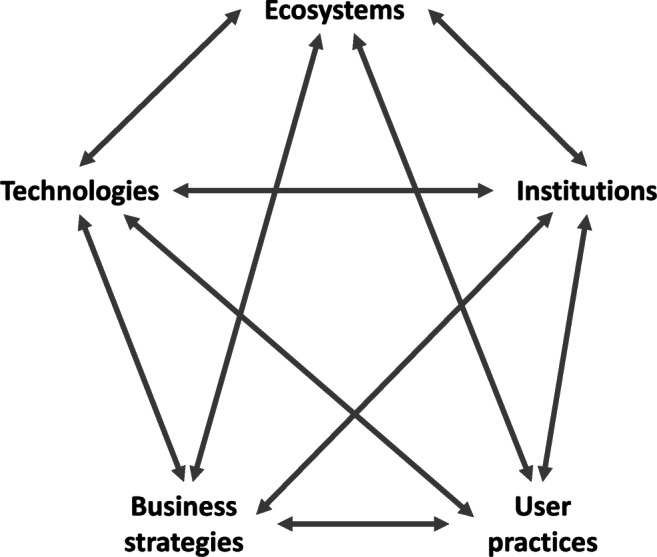
The co-evolutionary framework developed originally by Norgaard (1994) and adopted by Foxon (2011); Source: Foxon (2011)

Combining this line of thinking with the transitions management theory, Foxon (2011) developed a co-evolutionary framework to analyse the transition to a sustainable low-carbon economy (Foxon 2011). This framework is instrumental in analysing changes in ecosystems, technologies, institutions, business strategies and social practices by focusing on the long-term casual-effect relationships invoked by them as shown in Fig. 2 (Foxon 2011). Briefly, ecosystems refer to ‘systems of natural flows and interactions that maintain and enhance living systems’; technologies refer to ‘methods and designs for transforming matter, energy and information from one state to another’; institutions refer to ‘ways of structuring human/organisations interactions with the environment’; business strategies refer to ‘means and processes by which firms organise their activities so as to fulfil their socio-economic purposes’; and user practices refer to ‘routinized, culturally embedded patterns of behaviour relating to fulfilling human needs’ (Foxon 2011). As argued by the author, this type of analysis can be useful in overcoming lock-in to unsustainable high-carbon systems of production and consumption.

**Fig. 3:**
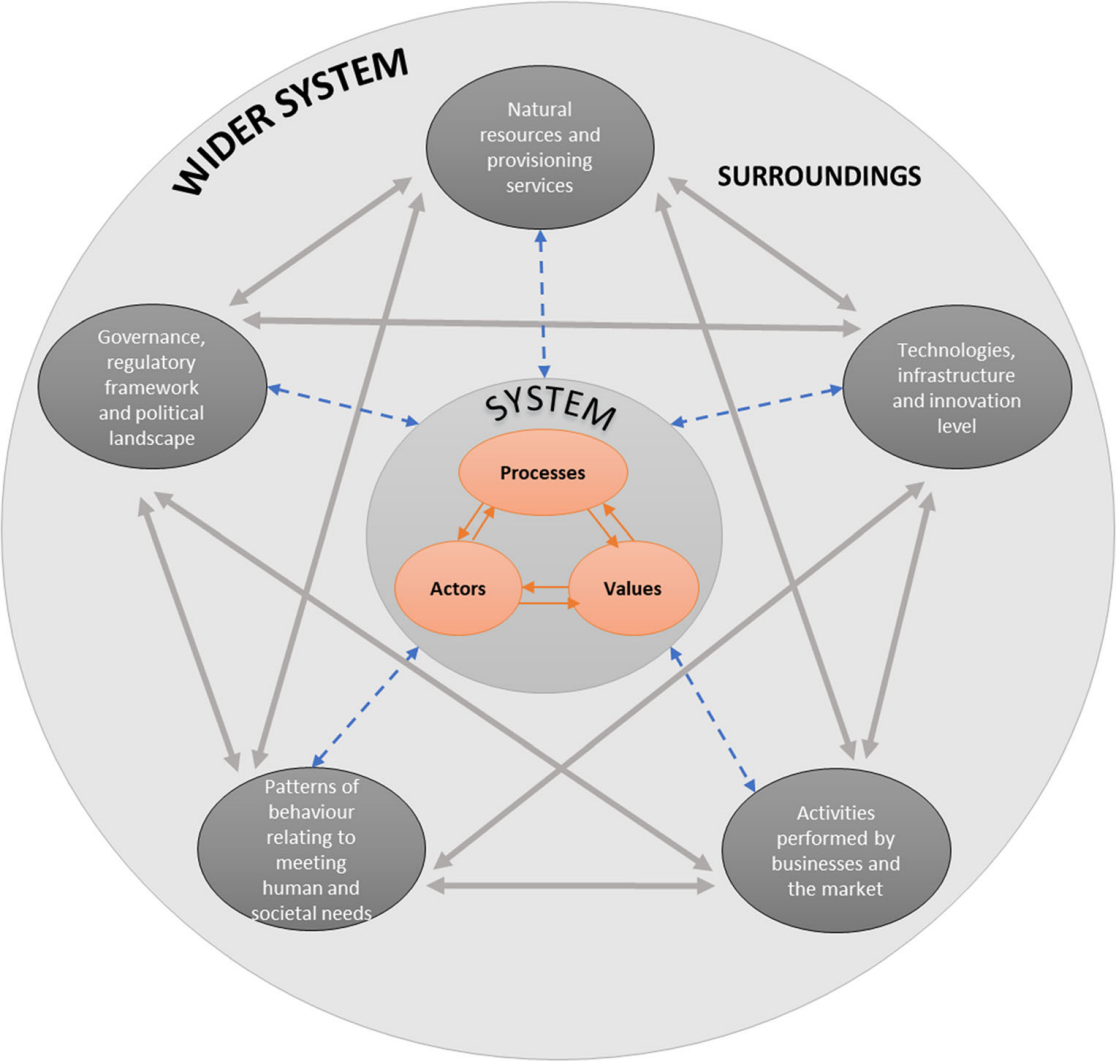
The resource recovery system with its internal sub-systems, as situated within the external sub-systems that form the whole system of systems

From an engineering perspective, this network of dynamically interacting sub-systems is called ‘System of Systems’ (SoS) (Abdoli et al. 2019). The SoS has been used since the 1950s to describe systems composed of independent constituent systems, or sub-systems, that synergistically interact towards achieving a common goal (Nielsen et al. 2015). Via synergism, sub-systems develop and respond to change interactively, which may impact on the success of system interventions. This signifies that the dynamic web of sub-systems that synergistically act together and affect one another through causality form the ‘landscape’ (other terms are ‘background’ or ‘enabling environment’); i.e., the space where the resource recovery system (and internal sub-systems) is situated in, and which casually affects the dynamics between processes, values and actors. This relationship is depicted in Fig. 3, where the sub-systems surrounding and casually affecting the resource recovery system are namely the following: *Natural environment and provisioning services* (as opposed to Ecosystems); *Technologies, infrastructure and innovation level* (as opposed to Technologies); G*overnance, regulatory framework and political landscape* (as opposed to Institutions); A*ctivities performed by businesses and the market* (as opposed to Business strategies); and *patterns of behaviour relating to human and societal needs* (as opposed to User practices).

**Fig. 4:**
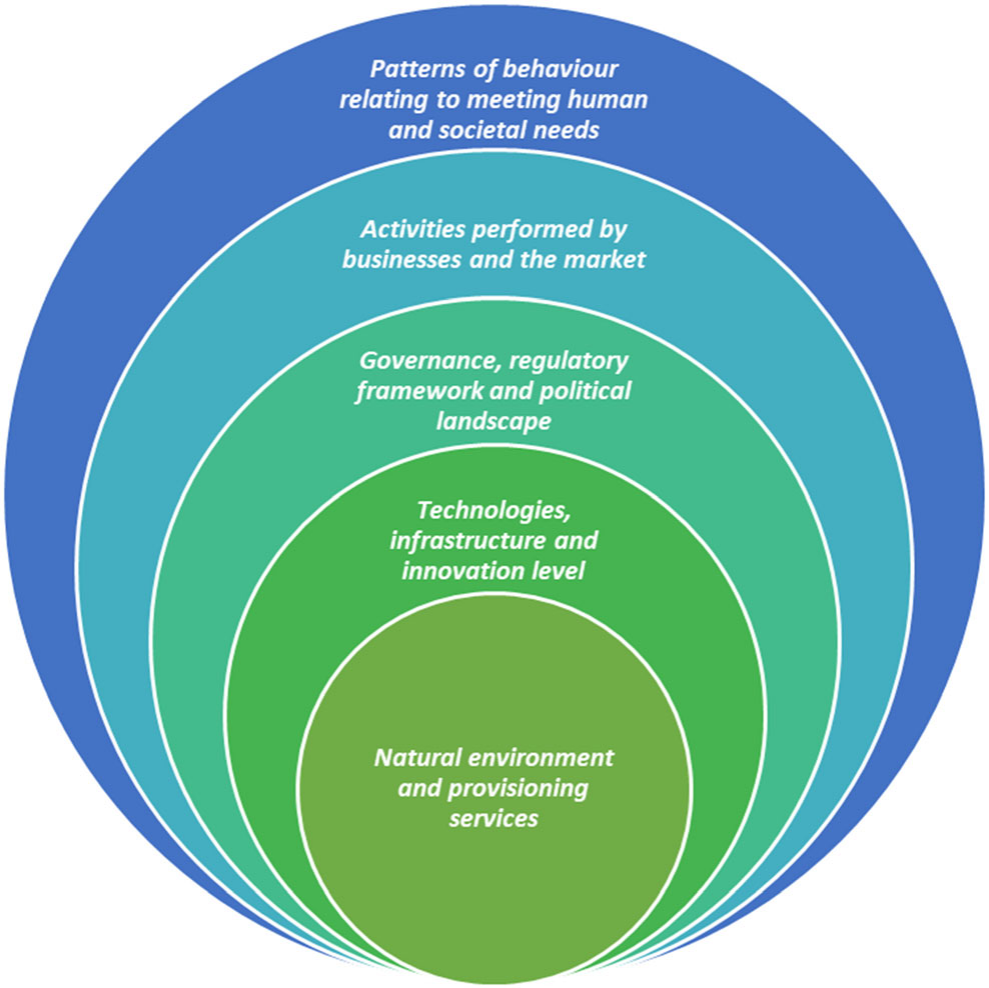
The ‘five levels of information’; a conceptual approach to understanding the dynamics, drivers and barriers of resource recovery systems; Source: (Iacovidou et al. 2020a)

The external sub-systems, illustrated in Fig. 3, can be perceived as interconnected entities attached to the resource recovery system or as the lenses through which the ‘enabling’ surrounding environment can be analysed as depicted in the Integrated Sustainable Solid Waste Management (ISWM) concept (Anschütz et al. 2004; Consortium 2009; Wilson et al. 2013). There is a direct (grey arrows) and indirect paths (blue dotted arrows) between any two of the external sub-systems and the resource recovery system (i.e. internal sub-systems), respectively. This illustrates that external sub-systems can affect the behaviour of the entire system directly and indirectly. They can, thus, be represented as layers of information to the internal, convoluted sub-systems (i.e. processes, actors and values), helping to interpret the way these (internal sub-systems) function, interact and change. Subsequently, we call these layers ‘levels of information’, because without knowledge on the relevant ecological, economic, social, political, institutional and organisational drivers of the wider system, no adequate picture can be drawn for assessing the resource recovery system. Also, they are called ‘levels’ because there is a tantalising, lenient hierarchy between them. The *natural environment and provisioning services* are closely linked with the inner system due to their effect on resource production, distribution and disposal itself. The *behavioural patterns* that underlie the socio-economic system can therefore be understood to ‘embrace’ the *provisioning services*, whilst the intermediate layers regulate, shape and filter these behavioural patterns into more concrete actions (e.g., transactions, information, exercise of influence) transforming them into the *provisioning services* under investigation. There might be a hierarchical order between the intermediary layers as well, but our framework has not evolved to a point at which we can make useful insights on this order. An initial insight is that their order in the wider system might be influenced by the specific material, component and product system and power dynamics of the stakeholders involved. The relation between the ‘five levels of information’ might thus be visualised as shown in Fig. 4.

The concentric approach of representing the ‘five levels of information’ provides a useful conceptual basis in guiding our understanding of the resource recovery systems behaviour. It can streamline the process of identifying the opportunities and barriers towards sustainable resource recovery, which in turn makes it possible to pinpoint where value is recovered, destroyed and dissipated in support of circularity. This depth and breadth of understanding complexity in the wider system context (Fig. 3) can be useful in generating insights into potential future interventions in the resource recovery systems. Whilst this approach is primarily developed to cut through the complexity of resource recovery systems, and promote informed transitions to sustainable, circular set-ups, we also acknowledge its potential to conceptualise important aspects in overcoming the lock-in to linear practices. In the following section, we use this conceptual approach to discuss challenges related to potential CE transitions.

## Using the ‘five levels of information’ to unpacking CE challenges

In this Section, we use the ‘five levels of information’ approach to unpack some of the CE challenges that we encountered in our research on promoting resource recovery from waste, with recent insights added to further support and validate our views. We acknowledge that there is now a plethora of papers discussing the various challenges of CE implementation from different perspectives. The degree to which some of these challenges are seen as barriers to circularity may vary depending on the type of resource recovery system and its spatial context. We do not attempt to make comparisons between different resources or contexts, nor is the purpose of this article. Instead, we use our systems-based conceptual approach to depict some of the resource recovery systems dynamics and drivers, and highlight some of the key challenges that currently prevent CE to become realised. We must stress that the following sub-sections do not aim to be exhaustive, as each level of information could be further subcategorized and investigated in depth.

### Natural environment and provisioning services

This level of information provides insights into the ecosystems impacted by resources consumption, production and management, and the role of provisioning services in supporting circularity. Human-derived (from nature) and designed MCPs and the way these are used and managed create many unknown risks for the environment and human health that may question the sustainability of resource recovery systems. Some potential risks were recently expressed in a few studies which are as follows: the use of woody biomass as biofuel may drive deforestation (Millward-Hopkins and Purnell 2019); plastics bottles may leach estrogenic compounds and cause adverse health impacts (Wagner and Oehlmann 2009; Yang et al. 2011); bio-based plastics are largely made from first-generation feedstock (i.e. food crops) of which production is associated with land-use change (Hottle et al. 2013; Piemonte and Gironi 2011) and competition for land required for food production (Álvarez-Chávez et al. 2012); biodegradable plastic waste that can be treated with organic wastes via composting or anaerobic digestion, may dilute compost’s or digestate’s nutritional value and instead become a neglected source of microplastics (Weithmann et al. 2018). Consequently, forcing MCPs into circular loops that are not well-understood in terms of their net sustainability benefit can adversely impact on our ecosystems and human well-being. To that end, correct establishment and terminology of physical flows and their cyclical ability are important challenges in enabling and supporting CE transitions based on net sustainability benefit.

Physical flows are defined as exchanges of matter and energy between the natural and societal production-consumption systems (e.g. air, fuel, energy and resource inputs, and wastes and emission outputs) and are culturally, temporally and spatially dependent (Akenji et al. 2016; Korhonen et al. 2018b). When the flows of MCPs are interrupted (i.e. accidental spillages, inappropriate collection, or mismanagement), these may leak to the environment via a number of sources and pathways. Some of the MCPs (e.g. technical nutrients, and even some biological ones) that leak to the environment may take years to degrade (e.g. plastics and bio-based alternatives), and as a result, they can cause serious harm to ecosystems and human health (Awasthi et al. 2019; Lau et al. 2020). Currently, there is a lack of consensus on what constitutes a source (i.e. origin of debris to the environment) and pathway (i.e. the route of debris from the point of emission to another point) due to varying perceptions around human behaviour, socio-economic aspects and socio-technical regimes in different areas (Iacovidou et al. 2020b). This lack of consensus limits our understanding on the way resources enter the various environmental compartments, and at which stage(s) in the system, restricting also our understanding on the point and way they intersect with other systems (e.g. microplastics entering the food chain) (Iacovidou et al. 2017b). Then there’s also the export of waste resources for recycling from one spatial system (usually a developed country) to another (usually a developing country), such as in the case of plastic waste and e-waste that are shipped from an EU country to a Southeast Asian country. Waste resources are rarely monitored once they cross borders, and as a result, their fate remains unknown. Yet, developed countries account the exported waste resources in their recycling estimations (and thus, circularity targets), whilst it is very likely that these wastes may be mismanaged (i.e., inadequately disposed and littered) contributing to negative value creation elsewhere with often deleterious impacts on ecosystems and human health (Huang et al. 2014; Iacovidou et al. 2020b; Lau et al. 2020). Foreign recyclable waste markets in developing countries tend to have a questionable waste infrastructure and often weaker, less reliable institutions to control the management of waste (Dauvergne 2018; Osibanjo and Nnorom 2007; Schmidt 2006).

Korhronen et al. (2018) suggest that in theory, it is possible to recycle everything using energy from the sun (Korhonen et al. 2018a). In practice, however, this is not currently feasible. Recycling relies largely on non-renewable energy, whilst it generates unavoidable waste and by-products that need further processing. Other waste management options that promote circularity, such as the reuse, repair and remanufacturing of components and products, can also lead to value negation. This is due to losses in quantity (physical material losses, by-products) and degradation of MCP quality (mixing, downgrading) (Cullen 2017). Varying degrees of virgin materials and energy are almost always injected into MCP ‘circular’ loops, which questions the net sustainability benefits of circularity (Korhonen et al. 2018a). Another challenge associated with achieving the CE is in regard to understanding the specificities of different MCP cycles, i.e. their flows and transformations in the system, including indications of ‘wear and tear’ and cascading effects (Iacovidou et al. 2019). Insights into the degradation of MCPs’ technical properties enable the selection of the waste management option that can return the maximum net sustainability benefit. Often, this may not be achievable via closing the loop, i.e. 100% material recovery (Geissdoerfer et al. 2017; Huysman et al. 2017).

The concept of closed loops, where MCPs remain in circulation and at high value, is in conflict with the widely used term of ‘waste’. This is a dynamic and controversial term that is contingent on time, culture, society, history, etc. (Desrochers 2002; Desrochers 2004; Pongracz 2002). In many developing countries, people are prepared to use functioning but superficially degraded components and products that people in developed countries might consider ‘waste’. This highlights the difficulty in deciding the exact moment that MCPs become waste, since they are entirely dependent on the social and cultural context in which they are placed. It is even obscure to distinguish between ‘waste’ and ‘by-product’. A significant fraction of post-industrial waste should be considered to be ‘by-product’ if treated within the same industry or via cross-sectoral collaborations between different industries that promote industrial symbiosis. For example, pulverised fly ash (PFA) from coal combustion or ground granulated blast-furnace slag (GGBS) from primary steel production are both process outputs that are considered by the parent industry to be essentially ‘waste’. These ‘wastes’ are widely used as ‘by-products’ in other industrial processes, particularly in concrete production by replacing 55 to 80% wt. of the required cement. The primary motivation of utilising these ‘by-products’ in the conrete production industry is to replace the most carbon intensive ingredient of concrete—cement—with a nominally zero-carbon (since the emissions associated with its production are attributed to the parent product, i.e. electricity or iron, see below) substitute (Millward-Hopkins et al. 2018b).

In regard to understanding the role of provisioning services in supporting the CE, distinctions between ‘wastes’ and ‘by-products’ are important to be made and attention must also be placed on emerging contexts, e.g. environmental thresholds and critical materials (Robèrt et al. 2013; Rockström et al. 2009), and the economic aspects of existing material capacity. Physical flows of materials and energy create both long-term and short-term environmental, economic, social and technical impacts, depending on how long they remain in the use phase (stocks) and the political and organisational aspects (Geissdoerfer et al. 2017; Ghisellini et al. 2016). This often impacts the dynamic and complex interdependencies of materials and energy flows (Millward-Hopkins et al. 2018a; Robèrt et al. 2013; Robèrt et al. 2002), leading to potential economic and organisational changes that may risk sustainability in the long-term (Davoudi and Sturzaker 2017; Korhonen et al. 2018a). Another implication is the lock-in situations where net sustainability benefits become blurred (Corvellec et al. 2013; Iacovidou et al. 2020a; Klitkou et al. 2015; Milios et al. 2018a). Using the previous example, coal-PFA and GGBS are currently considered low- or even zero-carbon materials. This makes their use as by-products in the concrete industry important, because it lowers the nominal carbon impacts of construction in addition to improvements in concrete’s performance, strength and durability (Millward-Hopkins et al. 2018b). It is argued that the use of PFA and GGBS as by-products should be allocated an amount of embodied carbon from primary production processes. Allocating GHG emissions in PFA and GGBS can de-incentivise the construction sector in utilising these by-products and may result in a transition from ‘by-products’ to ‘wastes’ that will be disposed of in landfills, leading to consequential impacts as well as primary resource consumption and emissions (Millward-Hopkins et al. 2018b). Non-allocation, however, incentivises coal-power producers to continue their high-carbon activities and offers an excuse to concrete producers and consumers to devote little attention to material efficiency (Millward-Hopkins and Purnell 2019). Respecting our ecosystems and well-being, and focusing on natural reproduction rates to the point nature can tolerate, is pivotal (Braungart et al. 2007; Korhonen 2001; Korhonen et al. 2018a)

### Governance, regulatory frameworks and political landscape

This information level scrutinises and filters the political aspects that underlie the socio- and techno-economic aspects of resource recovery systems. Governments around the world have a critical role to play in decision-making processes concerning the environment. Resources and waste management policy agendas have seen an increased popularity and development over the past decades. Other actors, such as businesses, industry, and NGOs, are also gaining critical decision-making roles in the field of resource efficiency (Ghisellini et al. 2016; Milios 2018). Whilst this is critical in advancing the transition to CE and creating the right capabilities of doing so (Geissdoerfer et al. 2018), it also reinforces siloed-thinking that may lead to sector-specific voluntary agreements and strategies for tackling issues at a specific stage in the value chain, or influencing a certain group of stakeholders (Williams 2019).

Sector-specific strategies (e.g. reduce plastic packaging used in food contact applications), like those promoted at the EU (European Commission 2018) and global level (Ellen MacArthur Foundation 2019), often disregard the implications of these actions on other sectors (e.g. the food system), leading to problem shifting in other system(s) (e.g. food waste spoilage). For example, raw fresh meat (without packaging) can last from 2 to 5 days, whereas the packaging can extend its life for up to 3 weeks. In developed countries, meat mass production involves a number of steps between slaughtering and consumption (e.g. storage, transportation, preparation and distribution to retailers). This signifies that more time is needed to safeguard the quality of the meat until it reaches the consumer, and the consumers decide to consume it. The latter is particularly important for consumers that want to have some flexibility on the meat consumption period from the date of purchase, without having to worry about compromising the quality of the product (FAO 2019). However, the plastic packaging used for extending the meat shelf-life is not currently recyclable, which can create negative implications to the plastic system. Nonetheless, it may reduce food waste at the retailer and household levels creating positive impacts on the food system (denkstatt 2017). Clearly, there are several trade-offs on the cross-over between different resource systems that need to be thoroughly examined and evaluated in order to identify the tipping points at which net sustainability benefits can be gained in each resource recovery system.

Moreover, strategies that seek to meet CE policy interventions occur at different implementation scales, namely the *macro*, *meso* and *micro* scales. According to the literature, *macro* refers to a city, province, region, or nation or it may take a global perspective; *meso* refers to eco-industrial parks, industries and companies; and *micro* refers to a single material, component or product, company, or consumer (Kirchherr et al. 2017; Moraga et al. 2019). The EU and EMF strategies focus largely on the *macro* and *meso* scales of implementation, usually manifested in the form of tangible targets (Morseletto 2020), neglecting to account for the specificities of different MCPs and way they influence the system as a whole (*micro* scale) (Iacovidou and Lovat 2021; Lonca et al. 2018). Targets provide direction, motivate action towards a predetermined direction, and require the commitment of the various stakeholders involved, whilst they monitor delivery of their intended outcome via a practical and measurable means (Akenji et al. 2016; Milios 2018; Morseletto 2020). Meeting the targets creates the illusion of achieving reductions in resource throughput and in improving waste management options’ circularity performance, whilst in fact it provides ‘snapshot’ circularity views of the system. This emphasises the need to look at the resource recovery system as a whole, from production to consumption and EoL management, and employ an approach that takes into account value considerations in other cross-linked systems. Whilst the basic idea of the CE is intuitive and convincing and the notion is widely used in policy documents, the assessment of progress towards a CE is an issue of ongoing debate.

The lack of system-wide tools and criteria for monitoring and measuring the circularity of MCPs presents a challenge to achieving a CE (Korhonen et al. 2018b; Willi et al. 2015). Several authors endeavoured to address this gap, pointing out to the importance of well-designed and effective indicators in the transition from linear to circular approaches (Di Maio and Rem 2015; Franklin-Johnson et al. 2016; Geng et al. 2012; Genovese et al. 2015; Guo-gang 2011; Huysman et al. 2017; Iacovidou et al. 2017b; Lonca et al. 2018; Moriguchi 2007; Park and Chertow 2014; Zhijun and Nailing 2007). Nonetheless, the lack of knowledge on how to make this transition without risking the financial and marketing values is fundamental (Schandl et al. 2015). Transitions management states that responses to address such a complex and uncertain goal should have five key characteristics (Rotmans et al., 2001):

- Policy-making based on long-term thinking;
- Considering of multiple domains and actors at different scales;
- Focusing on learning through experience;
- Fostering system innovation and system improvement;
- Avoiding lock-in, and keeping options open.

Policies and governmental interventions (e.g. via economic instruments) can be critical in promoting such practices. For example, the implementation of the extended producer responsibility (EPR) policies, which extends the producer’s responsibility for their components and products to the post-consumer stage of a product’s life cycle, is considered an efficient policy to improving the recovery of resources from waste (OECD 2014). EPR programmes are designed and implemented in different ways and may cover a range of products, e.g. packaging, batteries, EoL vehicles (ELVs) and EEE in the EU. Nonetheless, their efficiency is often hampered by the lack of transparency and adequate compliance control, price volatility, market competition, and other organisational and economic constraints (Campbell-Johnston et al. 2020; Iacovidou et al. 2020a). Laying down specific measures for establishing and maintaining a well-functioning market for recycled materials is mandatory for the successful implementation of such schemes. This requires consideration of the multiple value domains and the actors involved in order to orchestrate a move away from short financial and/or political cycles. The complexity of achieving this is extremely challenging, due to the perceived risks to businesses and the lack of value chain coordination amongst different actors that prevent costs and benefits from being equitably borne and appropriated.

Identifying useful interventions and exploring their knock-on effects on interconnected MCP systems, such as in the food-plastic packaging or the electricity-steel-concrete examples, is imperative in promoting resilience and sustainability in resource recovery systems. Technical, financial, environmental and social considerations of such systems should not be geographically divorced from the production-consumption-management processes. Using the UK as an example, decarbonisation transitions have made PFA and GGBS by-products increasingly unavailable or unsuitable, presenting a challenge for the concrete producers. To address this challenge, PFA and GGBS may be imported into the UK from other countries, rapidly turning PFA and GGBS into internationally traded commodities (Millward-Hopkins et al. 2018b). This seems beneficial in terms of resource recovery, but it may incentivise unsustainable practices in countries that are vulnerable to risks including particulate emissions, thereby leading to problem shifting elsewhere in the system.

Furthermore, there’s also trade-offs associated with the national and international efforts to promote resource efficiency and sustainability. These may be environmental, e.g. when policies for low-carbon power increase biofuel demand, which, in turn, reduces carbon sequestration in some forest types (Hudiburg et al. 2011), or when land-use change for meeting global food demand damages biodiversity and leads to deforestation (Carrasco et al. 2017). They may be political, e.g. when growing demand for food leads to a demand in agricultural land and intensifies conflicts over land-use (Scheidel and Sorman 2012). They may also be economic, e.g. when higher resource prices and exports contribute to an appreciation of the exchange rates (Windle and Rolfe 2014); and social, e.g. when recycling targets in ‘developed’ countries may displace risks (via exports of waste, e.g. plastics and e-waste) in importing countries (Velis 2017), or when land-use change alters ecosystems, which in turn can increase the interaction of wild animals with humans and expose humans to new pathogens (White and Razgour 2020).

Consideration of such complexities adds to the already complex decision-making processes that underpin CE transitions. Even so, these considerations are useful for supporting policy decisions and investments ahead of a highly uncertain future; a conceptually mundane proposition, which remains notoriously difficult to implement (Millward-Hopkins et al. 2018b).

### Activities performed by businesses and the market

This information level concerns the organisational relations that cause and drive resource flows through the system in order to meet the respective human and societal needs. It generates insights into the way economic incentives, market stability and information flow drive the activities of the businesses, and impacts on the wider system. Economic growth has evolved in such a way as to associate human well-being with growing resource consumption (Schandl et al. 2015). The long-standing reinforcement of this association through advertising, lobbying and political acceptance has led to a growing overconsumption ethos that has created numerous problems. The greatest problem is that secondary resources are not exploited in a sustainable manner; thus, their redistribution back to the economy is cascaded (or else downgraded).

The increased price volatility of secondary resources because of political situations and unpredictable market conditions and other events (e.g. related to climate change, pandemics) hinders their absorption by the market (Nicolli et al. 2012). Volatile markets for waste products can reduce the expected revenues associated with operating a waste treatment facility and the expected investment returns, hence diminishing recycling activities. In addition to that, secondary resource markets are highly fragmented due to the types, locations and amounts of materials salvaged (often not matching demand), and this hampers the development of proper mechanisms that would help establish the secondary resource supply chain (Genovese et al. 2015; Nicolli et al. 2012). Asymmetric information in regard to the quality of recyclable materials is also obstructing potential increases in recycling activities. Buyers (e.g. reprocessors, manufacturers) often bear the burden of generating the proof that the recyclable materials that enter their manufacturing processes are of sufficient high quality (Milios et al. 2018b). Moreover, secondary materials are in direct economic competition with low-priced and/or stably priced virgin materials, generating economic risk and negatively affecting investments in the recycling industry (Milios et al. 2018b), making it difficult to implement a transition to alternative business practices.

Current business models focus almost exclusively on the return on financial investment. The way businesses sell and distribute their services, components and products to the market is of utmost importance to them (Ritzén and Sandström 2017), and very few take into account design aspects and responsibility for the management of their components and products at their EoL stage. Moreover, many products may be deliberately designed with limited product life, as well as with limited upgradeability and reparability (Linder and Williander 2017). This helps businesses to avoid market saturation, and retain longevity and financial stability, a phenomenon known as planned obsolescence (Wieser 2016). A negative consequence of planned obsolescence is waste generation and a continued and often increased raw material extraction. Progress to reduce planned obsolescence appears to be slow, and often, producers claim that obsolescence can be a side effect of their efforts to ‘improve user-friendliness, lower prices or develop new technologies’ as explained and discussed in Maitre-Ekern and Dalhammar’s study (2016). This points to the fact that the development and enactment of strategies must be manifold in order to suit different business types (Rizos et al. 2016), different MCPs, and styles of operation, to help businesses re-establish their business models.

A growing number of businesses are striving to adopt strategies that support them in enhancing their environmental and financial performance, and accountability, but many of these efforts are as Jones and Comfort (2017) suggest ‘aspirational’ (Jones and Comfort 2017). Increased effort to overcome operational constraints in integrating sustainability considerations on the organisational level and improving resource productivity has led to the development of circular business models (CBM) (Geissdoerfer et al. 2018; Linder and Williander 2017; Rosa et al. 2019). These models have been around for decades, such as the product-service systems (Stahel 1994) and reverse logistics (Hosseini et al. 2015). Their operating principle lies on retaining the economic, environmental and social value of MCPs via the adoption of strategies that can prolong the useful life of components and products (e.g. reuse repair and remanufacturing) and can close material loops (e.g. recycling) (Bocken et al. 2016; Nußholz 2018; Ranta et al. 2018; Rosa et al. 2019). Many of these could support a move to a service-based rather (as opposed to product-based) business model, where contracts between suppliers and users will be based on continued supply of a given utility instead of a commodity or product (e.g. warmth and comfort instead of fuel, personal mobility instead of cars or flight time instead of aero engines) (Roelich et al. 2015). Examples of such strategies are well-discussed in (Bocken et al. 2016). The adoption and successful implementation of CBMs remains underexplored (Lewandowski 2016); and is associated with considerable obstacles (e.g. lack of supply and demand networks, lack of appropriate policy instruments, financial capacity) (Rizos et al. 2016), and risks (e.g. financial stability, technical, functional and economical vulnerability) (Linder and Williander 2017). This calls for reforms in national and international policy to enable the development of a new landscape that fosters collaboration between value chain stakeholders that are willing to adopt a new way of doing business, and which creates new value networks that can support CBM development and implementation.

Subsequently, the alignment of businesses’ priorities with those at the policy spheres and the introduction of policy mechanisms that can promote radical innovation and change in the way MCPs are used, recovered, stored, inventoried and returned back to the system (market) are gradually gaining traction by some businesses and policymakers within Europe for retaining resources in economic use for as long as possible (Hill 2015). Achieving a balance between supply and demand of secondary resources will construct a strong foundation for businesses growth and investment, elucidating the value of these secondary resources, and its appropriation and distribution amongst all stakeholders (Iacovidou et al. 2017a; Velenturf and Purnell 2017). This demands a mix of innovative concepts and actors, prepared and incentivised to take long-term risks (Seuring 2004; Seuring and Gold 2013); designers and intermediaries who can make appropriate radical changes in processes and practices; and decision-makers that are willing to use new valuation tools (Golinska et al. 2015; Küçüksayraç et al. 2015).

### Technologies, infrastructure and innovation level

This level of information concerns the technological and infrastructure elements that are integral part of resource recovery systems and have an important role to play in promoting CE. The most striking feature of MCPs is that their properties begin to degrade once they enter the technosphere (Ayres 1999; Converse 1996, 1997; Craig 2001; Korhonen 2001). The degradation of MCP properties intensifies whilst they are in use, and during their disposal and management stages (e.g. shredding of functional products or cross-contamination between materials), and as a result, their return back to the supply chain is never entirely circular. For example, recycling promotes the production of secondary resources with deteriorating attributes, e.g. plastics and paper, to be used as feedstock in the production of new and inferior products, so-called downcycling. Losses may also occur, owing to the methods used for collection, sorting and processing of materials. Thereby, the greatest challenge of CE is to devise ways to delay the degradation process of MCPs in the value chain and prevent their ‘cascading’ into waste.

At present repair, remanufacture and reuse options are suppressed by the lack of supply and demand networks of second hand components and products. Instead, a waste-centric system prevails which is run by well-established technologies (e.g. energy from waste, and mechanical biological treatment for RDF/SRF production, recycling, landfill). The increasing financial return and the vast experience with the use of the existing well-established technologies that offer competitive advantages over competing technologies, create a sense of security, which effectively maintain a technological lock-in (Iacovidou et al. 2020a). In developing countries, the infrastructure is suboptimal, and this may be due to regulations that are often misused to benefit powerful stakeholders that may not be interested in investing to new waste infrastructure, or due to the lack of organisational capacities, financial resources and cultural aspects (Guerrero et al. 2013). Much of the ‘heavy lifting’ in these contexts is carried out by the informal sector, which involves scavengers or waste pickers that provide waste management services in the street, storage sites and at dump sites/landfills where there is no formal waste management system in place (Sembiring and Nitivattananon 2010; Wilson et al. 2006). There are multiple economic and social positive and negative impacts associated with the informal sector which are well-described elsewhere (Medina 2000; Wilson et al. 2006). The challenge in such settings is often the lack of cooperation between the informal and formal sector that hampers development and innovation in resource recovery systems.

Re-designing MCPs in a way that promotes longevity by repairability and disassembly and product upgradability (Masi et al. 2017) can then shift efforts towards maintaining their functionality and value in the system, developing also a clear recovery route. But how often do designers take such considerations on-board? Perhaps, rarely. Even in the construction sector, where off-site pre-fabrication of modular components, e.g. beams, columns, occurs for assembly on-site, is promoted as a way to improve resource efficiency (Lawson and Ogden 2010), EoL considerations of modular components are currently outside its remit. This signifies that innovation is growing in silo, and is not always (if ever) aligned with investments and innovation upstream and downstream of MCPs’ value chains. This, in turn, highlights that innovation and competition can often be detached from CE principles.

Innovations in the design of MCPs should be ratified only when the EoL considerations of MCPs are taken into account. However, current practices suggest that this is rarely the case. The sustainability of recent innovations, such as the design of components and products from alternative materials (e.g. bioplastics), or with an increased recycled content, and others that are material and carbon efficient (e.g. aluminium-made cars), is increasingly debated (Bakker et al. 2014; Winkler 2011). Even if innovations at the design stage are made with a consideration of the EoL management, would these denote that the right infrastructure is actually in place to support sustainable management and CE? Take for example, the use of compostable bags. These plastic bags can only be composted at in-vessel composting. If in the target area, there are only open-air composting facilities, the use of compostable bags will add complexity to the plastic management problem, rather than provide a solution. Designing repairable and upgradable products, without placing the right means and skills to support their EoL management, is nonsensical. Which points to the fact that efforts to redesign components and products to improve their sustainability performance and management would need to be supported by policy instruments (e.g. take-back schemes, information- and market-based instruments) and infrastructure. Infrastructure requires investment, a stable political framework, suitable markets for reclaimed components and products, and a predictable supply of components or products to ensure that such venture would be viable and profitable (Iacovidou et al. 2018).

Nonetheless, the lack of traceability of components’ and products’ characteristics and performance in the value chain is a key barrier in promoting their recovery and circularity at their end-of-use (EoU) stage. This not only hinders investment in infrastructure (Iacovidou et al. 2018) but also prevents CBM to become realised. Efforts to address this barrier have led to the emergence of product life cycle management (PLM). PLM is broadly defined as the knowledge management solution in which product data are shared amongst stakeholders and processes at the different phases of the product’s life cycle via the support of information and communication technologies (ICT). It integrates processes and actors through knowledge; it monitors that the desired performance is attained throughout the product’s life cycle (hence improving product and service quality); it ensures that the product is sustainably managed; and, finally, it enables robust decision-making at the design stage (Ameri and Dutta 2005; Terzi et al. 2010). In PLM, technology has an active role. It helps to create the information (needed to create the knowledge) and helps to store and transfer this information throughout the product’s life cycle.

Advanced ‘smart’ technologies including radio frequency identification (RFID) tags, optical character recognition, 3D scanning laser, building information modelling (BIM), sensor networks, blockchain technology are important tools in promoting the traceability and recovery of components and products in many sectors and essentially supporting circularity (Feng 2016; Iacovidou et al. 2018; Pan 2016). For example, RFID can be used to track and store information about a component’s properties over time, it is ease to handle and it is durable and affordable (Lim and Winsper 2012; Majrouhi Sardroud 2012). Integrating RFID with BIM can be an innovative solution in the construction sector, enabling reclaimed construction components to be tracked over their lifetime, changes in their properties archived, promoting reuse into new structures when they reach their initial EoU stage (Iacovidou et al. 2018).

Knowledge creation can lead to innovation and collaboration amongst stakeholders. Innovation, investment (e.g. infrastructure and digitalisation) and training (for skills development) that can promote the reconditioning, upgrading and reuse of MCPs will conversely minimise their ‘cascading’ in the resource recovery system. This will create opportunities to develop holistic solutions that integrate upstream and downstream practices and effectively promote circularity. The challenge with the technologies that are currently used to transform information and knoweldge, and promote the life cycle management of MCPs is the lack of trust and capability of employing them the first place. Progress in this area is currently slow, but is likely to be gaining traction soon, due to rapid technological development supported by Industry 4.0, and the growing attention in CBM development (Chertow and Ehrenfeld 2012).

### Patterns of behaviour relating to meeting human and societal needs

This level of information refers to the importance of behavioural patterns that are evolved over time as a result of meeting basic human needs (e.g., for food) and the human ability to organise socially in order to improve the provisioning of the means to cater for such needs. Consumers have a pivotal role to play in the transition to CE as their lifestyle choices (affected by their cultural background, status, education, etc.) are increasingly shaped by environmental considerations; yet global capitalism and consumerist lifestyles grow even more demanding on the environment, whilst there is a ‘tendency for individuals to see themselves as separate from nature’ (p.1081) (Schultz 2011). This has led to a growing throwaway society that puts more pressure on natural resources and devaluates recycled and reused resources (Williams 2019). As Williams (2019) points out, ‘existing systems of provision tend to reinforce this throwaway culture by operating linearly’ (p.5). For example, the products that are placed on the market, the rate at which they are placed, the reduction of household waste, and food waste in particular, and the sorting of waste in the household depend on human behaviour, habits, choices and values.

Consumers’ behaviour is guided by their habits, and their habits influence their choices, which are further driven by external factors such as brand, the aesthetic qualities of products and the price, all of which can be important factors in determining consumers’ perception, preference and purchasing decisions (IEEP 2018; Jones and Comfort 2017; Maréchal 2010). Likewise, manufacturers and brand designers are driven by the consumers’ buying behaviour, and they are ‘forced’ to make their products to stand out in order to gain a competitive edge on the marketplace. As a result, excessive printing and branding on consumer products are often amplified to make a product attractive to the consumer (Iacovidou et al. 2019). Consumers’ choices have the power to change the current regime, by showing preference to less excessively advertised, and yet helpfully labelled, products. This can drive innovation in new business models’ development and technologies, which in turn can lead to the development of ‘new’ MCPs.

Jones and Comfort (2017) suggest that using instruments, such as incentives (e.g. money return when using reverse vending machines, lower prices in products, heat for free), can help consumers change their habits. Incentives can be more effective than regulations that try to ensure that prices are adequately reflected in products. Maréchal (2010) suggests that incentives can be effective in changing habits (and ultimately behaviour) only if the right information is given to consumers and can be efficient only when consumers present a high receptivity to the new information (Maréchal 2010). This underlines that the transition to CE depends on creating and communicating the right information that will urge consumers to implement changes in the way they purchase and use products and services, and on the way they dispose them at their EoU stage, overcoming the ‘lock in (and provide ways out of) particular ways of doing things’ (Moloney and Strengers 2014). In regard to disposal, the littering problem has gained growing attention recently, as a result of the marine plastic pollution. Since, a large proportion of marine plastic waste comes from land-based sources, raising consumers’ awareness in order to act upon it has been considered a suitable strategy to minimise the problem (Jefferson et al. 2015).

Henderson and Green (2020) point also to the fact that the many products that consumers use contribute to microplastics (particles under 5 mm in length) production, mainly through the use of plastic components and products (in different applications/sectors), textiles, tyres that contaminate environmental compartments and the marine food chains (Henderson and Green 2020). They highlight that often, social media, to which consumers rely on to get informed on different societal aspects and get help with their purchasing decisions, fail to convey the right messages and prevent consumers from changing their purchasing and consumption habits (Henderson and Green 2020). Consumers are also influenced by their cultural backgrounds, education level/knowledge, circumstances, beliefs, social practices and values, all of which points out that for media to effectively communicate with consumers on the various aspects pertinent to the use of different MCPs, it needs to be scientifically comprehended, rigorous, and responsive to cultural specificities and attitudes (Henderson and Green 2020). Media, educational and public awareness campaigns, have a central role to play in shaping public understandings and in promoting responsible environmental attitudes to consumption (Henderson and Green 2020; Williams 2019). In turn, this will ensure that efforts in the social-economic-political domains are well-aligned and will help to unlock many benefits for the society such as preference to local businesses that could create local jobs and provide opportunities for social integration and cohesion.

Changing the cultural mindset and practices, however, can create variability in the way people behave towards environmental responsibility, which may or may not lead to the successful transition to a CE. Take for example components/ products ownership. According to Maréchal (2010), people can display behavioural lock-in, which may result to a change-resisting nature that may contribute to maintaining established consumption/use practices (Maréchal 2010). This suggests that a system, in which products are provided as services (e.g. product-service system), could be slow in becoming mainstream, as people will be less likely to become detached from MCPs and focus more on the utility that these provide. What’s more, to enable change, consumers may want to feel rewarded for their efforts in promoting sustainability in the system, and businesses will need to provide the right incentives and information in order to establish and maintain progress (Moloney and Strengers 2014).

Moreover, incentivising consumers to do the right thing may also help them become aware that MCPs that are useless to them can have an important value to others. This increased awareness can help consumers to appreciate the value embedded in MCPs and realise that their proper recovery and reintroduction back to the economy in the form of secondary/second hand MCPs can create benefits to the environment, economy and society. This links back to the fact that information is both critical and crucial in helping consumers’ make the right decisions, and becoming more mindful with the way they use and dispose/segregate their MCPs in the household and on-the-go. The more aware the public becomes of the social and personal benefits of adapting new practices, the more willing they will be to maintain their efforts; hence creating a *domino effect* in changing consumerist, throwaway lifestyles and habits.

The establishment of regional CEs via the product-service value chains can help communities grow and become partly self-sufficient and resilient. In the product-service value chains the costs of disposal and recovery are transferred to the producers who will be strongly incentivised to supply functionalities rather than components and products, ensuring that there is minimal transfer of resources into waste streams, e.g. by reconditioning products (Mancini 2011). In support of that idea, Connett et al. (2011) report that if a product cannot be reused, recycled or composted, then it is likely that it should not have been produced in the first place (Connett et al. 2011). This links back to MCPs’ functionality, service life and ownership, key variables in the power relations between stakeholders, the operation of the market, implementation of sustainable innovation and in the effectiveness of new policy measures that could foster the transition towards a sustainable CE.

## Conclusions

CE is associated with numerous challenges, most of which are not easy to overcome as they are deeply embedded into the way current regimes operate. Even though numerous attempts and progress have been made in many areas to decouple economic growth from human well-being and promote resource efficiency, remnants of past and present linear practices continue to haunt and prevent our efforts to change the conventional processes, structures and institutions that govern our resource recovery systems. To break this lock-in and realise the transition to CE, we need to accept that circularity is not always synonymous with sustainability and that CE is an ideal that we should pursue within the context of sustainability. We urgently need to move away from a waste-centric system, where MCPs’ quality degrades as they flow through the production, consumption and management systems. Radical changes at the design stage, technological advancement and innovation at the all stages, and a change of mindset at the consumption and management stages are all urgently needed to significantly lessen the amount of value dissipated in the MCP system. The analysis in the ‘Using the ‘five levels of information’ to unpacking CE challenges’ section highlights that the challenges of achieving CE described at each of the ‘five levels of information’ are quite interlinked. Accordingly, addressing these challenges requires a holistic approach that ensures solutions at one system (or at one point of the system) will not be creating problems at another system (or at another point of that same system); an apporach that will be tackling problems at their roots. We have shown that a system of systems approach is an important means to respond to this ultimatum, by examining and assessing the internal and external sub-systems elements and interrelationships, and by understanding how a resource recovery system evolves culturally, temporally and spatially.

Systems thinking is embedded in many of the existing theories, ontologies, concepts and tools that are currently being used by different disciplines to address the importance of overcoming obstacles to realising change and making the transition to a circular, sustainable future. We have tried to bring some of these together in creating a unifying approach of assessing our resource and waste systems. We believe this amalgamation is pivotal in overcoming disciplinary barriers and in promoting a truly transdisciplinary approach in grasping the multi-faceted political, environmental, economic, social and technical impacts associated with the production, use and management of resources. Such analytical scrutiny, which requires forward-thinking and time-investment, is more likely to illuminate suitable hotspots for future interventions and lead to the development of well-targeted transitional policy measures and socio-economic instruments (e.g. taxation, financing, incentives, governance mechanisms, communication and awareness raising tools). A systems-based approach that can transcend disciplinary boundaries can be extremely powerful in enabling well-informed and meaningful decision-making processes that challenge current norms, values and practices. Further research is needed to assess and evaluate the challenges and trade-offs of circularity transitions and make informed decisions as to what needs to be changed and how to best support that change via the development of suitable policies and tools.

## Data Availability

Not applicable

## Abbreviations

CBMs: Circular business models
CE: Circular economy
COTREP: Center of Resources and Expertise on the Recyclability of Plastic Household Packaging in France
CVORR: Complex value optimisation for resource recovery
EEE: Electric and electronic equipment
EoL: End-of-life
EoU: End-of-use
EU: European Union
EVOH: Ethylene vinyl alcohol
FSSD: Framework for Strategic Sustainable Development
GGBS: Ground granulated blast-furnace slag
GHG: Greenhouse gas
IOA: Input-output analysis
LCA: Life cycle assessment
MLP: Multi-level perspective
MCPs: Materials, components and products
PET: Polyethylene terephthalate
PE: Polyethylene
PP: Polypropylene
PFA: Pulverised fly ash
SiOx: Silicon oxide
SoS: System of Systems
TIS: Technological Innovation Systems
